# Upper and Lower Airways Evaluation and Its Relationship with Dynamic Upper Airway Obstruction in Racehorses

**DOI:** 10.3390/ani12121563

**Published:** 2022-06-17

**Authors:** Chiara Maria Lo Feudo, Giovanni Stancari, Federica Collavo, Luca Stucchi, Bianca Conturba, Enrica Zucca, Francesco Ferrucci

**Affiliations:** 1 Equine Sports Medicine Laboratory “Franco Tradati”, Department of Veterinary Medicine and Animal Sciences, Università Degli Studi di Milano, 26900 Lodi, Italy; chiara.lofeudo@unimi.it (C.M.L.F.); enrica.zucca@unimi.it (E.Z.); 2 Veterinary Teaching Hospital, Department of Veterinary Medicine and Animal Sciences, Università Degli Studi di Milano, 26900 Lodi, Italy; giovanni.stancari@unimi.it (G.S.); luca.stucchi@unimi.it (L.S.); bianca.conturba@unimi.it (B.C.); 3 Freelance DVM, Rheinbacher Straße 24, 53919 Weilerswist, Germany; federicacollavo@gmail.com

**Keywords:** DUAO, equine, horse, dynamic upper airway obstruction, upper respiratory tract, equine sports medicine, equine endoscopy, upper airway endoscopy

## Abstract

**Simple Summary:**

Dynamic upper airway obstructions (DUAO) are a common cause of poor performance in racehorses, including different forms which vary in severity. Previous studies reported contrasting results concerning the contribution of abnormal pharyngo-laryngeal appearance and airway inflammation to the pathogenesis of DUAO. The present study aimed to evaluate possible associations between the development of DUAO and resting airway endoscopic findings, epiglottis size, airways inflammation and exercise-induced pulmonary hemorrhage (EIPH). These relationships were statistically investigated retrospectively in 360 racehorses (Standardbreds and Thoroughbreds) with poor performance or abnormal respiratory noises. A flaccid appearance of the epiglottis was associated with the occurrence of the dorsal displacement of the soft palate, while no relationship was detected between DUAO and epiglottis length. Inflammation of the upper and lower airways was not related with the development of DUAO, nor were horses with DUAO more prone to experience EIPH. These results suggest that epiglottis may contribute to upper airway stability, while inflammation does not predispose horses to the onset of DUAO.

**Abstract:**

Dynamic upper airway obstructions (DUAO) are common in racehorses, but their pathogenetic mechanisms have not been completely clarified yet. Multiple studies suggest that alterations of the pharyngo-laryngeal region visible at resting endoscopy may be predictive of the onset of DUAO, and the development of DUAO may be associated with pharyngeal lymphoid hyperplasia (PLH), lower airway inflammation (LAI) and exercise-induced pulmonary hemorrhage (EIPH). The present study aims to investigate the possible relationship between the findings of a complete resting evaluation of the upper and lower airways and DUAO. In this retrospective study, 360 racehorses (Standardbreds and Thoroughbreds) referred for poor performance or abnormal respiratory noises were enrolled and underwent a diagnostic protocol including resting and high-speed treadmill endoscopy, cytological examination of the bronchoalveolar lavage fluid and radiographic assessment of the epiglottis length. In this population, epiglottis flaccidity was associated with dorsal displacement of the soft palate, while no relationship was detected between DUAO and epiglottis length. No associations were detected between DUAO and PLH, LAI or EIPH. In conclusion, it is likely that epiglottis plays a role in upper airway stability, while airways inflammation does not seem to be involved in the pathogenesis of DUAO.

## 1. Introduction

Horses are obligatory nasal breathers and cannot avoid the high pressures occurring at the level of the nasopharynx during exercise by switching to oral breathing like other species. Moreover, the nasopharyngeal region is not supported by osseous or cartilaginous structures and relies only on muscle activity to maintain its stability [1,2]: therefore, during exercise, when airflow turbulence and negative pressures occur at the floor of the nasopharynx and within the larynx, these structures may collapse, and horses may develop different forms of dynamic upper airway obstruction (DUAO) [3]. As a consequence, respiratory function and gas exchanges at the alveolar-capillary level may be impaired, determining poor athletic performance, especially in racehorses working at supramaximal exercise [4,5,6,7].

Different types of DUAO have been described, including dorsal displacement of the soft palate (DDSP), medial deviation of aryepiglottic folds (MDAF), nasopharyngeal collapse (NPC), dynamic laryngeal collapse (DLC), epiglottis entrapment (EE) and epiglottis retroversion (ER) [8]. Although these conditions have been studied for a long time, their exact pathogenetic mechanisms are still not completely clarified and are likely to be multifactorial [8,9,10]. Most DUAOs are thought to result from neuromuscular dysfunction, fatigue, immaturity or conformational change of the structures controlling the patency of the nasopharyngeal region [8,9,11]. In particular, multiple researchers have hypothesized that epiglottic conformation may play an important role in the stability of upper airways [12]: in fact, the loss of epiglottis convexity has been associated with the development of DDSP [12,13] and MDAF [12,14] and is frequently reported as a flaccid appearance of the epiglottis. Abnormal epiglottis conformation was associated with lower earnings in young racehorses [15]: this result could be attributable to the development of DUAO in horses with a dysplastic epiglottis. Another study reported improved performance in horses after epiglottic augmentation, which may overcome deficits in neuromuscular function [16]. However, the findings of other studies do not support this hypothesis, as they failed to find an association between a flaccid appearance of epiglottis at rest and the detection of DDSP during exercise [17]. Moreover, some authors proposed that a short epiglottis, measured radiographically, may contribute to the pathogenesis of DDSP [18], but other studies reported normal epiglottis length in horses with DDSP [9,19]. Therefore, it is still debated whether a functional epiglottis with normal conformation is required to maintain soft palate stability. Other nasopharyngeal abnormalities, visible at resting endoscopy, have been proposed as predictors of DUAO, including episodes of DDSP, which can occur spontaneously or be induced by swallowing, and the presence of ulceration on the free margin of the soft palate [9,20,21,22]. Spontaneous DDSP during resting endoscopy has been reported as a highly specific but extremely insensitive test for DDSP during exercise [23]; however, the clinical significance of DDSP at rest remains uncertain, as it can also occur in horses with a normal function of the upper airway during exercise [17,22]. Palate ulceration may occur due to the friction between the ventral surface of the epiglottis and the free border of the soft palate [16]; however, in a study, only 16% of the horses with DDSP had palate ulceration [24], and vice versa—many horses with palate ulceration do not show DDSP during exercise [17]. It has been hypothesized that horses displacing quickly do not have enough abrasion between the epiglottis and palate to induce ulceration [16].

Different authors reported that inflammation of the nasopharyngeal region, visible at endoscopy as pharyngeal lymphoid hyperplasia (PLH), may impair the function of pharyngeal mechanoceptors [25] and contribute to the neuromuscular dysfunction and instability of the upper airway, predisposing horses to the onset of DUAOs, such as DDSP, NPC and ER [8,9,26,27,28,29,30]. Recently, the “one airway, one disease” concept, long known in human medicine and describing a relationship between the health of the upper and lower airways, has also been proposed and investigated in equine medicine [31]. Lower airway inflammation (LAI) has been associated with DUAO [9,28,30,31,32,33], probably because of the increased negative pressure driven by lower respiratory tract obstruction and increased respiratory impedance and work of breathing, which may impair upper airway patency and accelerate the onset of neuromuscular fatigue [30,31,33,34,35]. Another proposed theory is that DUAO may predispose horses to lower airway disease via unknown mechanisms [9,33]. Another lower airway disorder that has been associated with DUAO is exercise-induced pulmonary hemorrhage (EIPH) [9,32,36]. This may be due to the increase in the transmural pulmonary capillary pressure gradient resulting from upper airway obstruction and leading to the rupture of pulmonary capillaries and therefore EIPH [1,31,36,37]. In contrast, no association between DUAO and LAI inflammation or EIPH was detected in another study [38].

As the role of upper and lower airways resting evaluation has not been fully understood yet, the present study aims to investigate, in a wide population of racehorses, the clinical significance of upper airway anatomical and functional resting abnormalities and the possible contribution of upper and lower airway inflammation to the development of DUAO.

## 2. Materials and Methods

### 2.1. Horses

The clinical records of Standardbred and Thoroughbred racehorses referred to the Equine Sports Medicine Unit of the Veterinary Teaching Hospital of the University of Milan (Italy) between 2000 and 2021, with a history of poor performance or abnormal respiratory noises during exercise, were retrospectively reviewed. All horses (*n* = 366) were in full training upon admission. Each horse underwent a complete clinical examination, laboratory analyses and upper airway endoscopy at rest and during exercise on a high-speed treadmill. In 158 horses, the length of epiglottis was measured radiographically. In 339 horses, tracheobronchoscopy was performed 30 min after maximal exercise on a treadmill for EIPH evaluation. In 297 horses, lower airway endoscopy was performed at rest, at least 24 h after exercise, and bronchoalveolar lavage fluid (BALf) was collected in 265 horses and subjected to cytological examination.

### 2.2. Upper Airway Endoscopy at Rest

Following a complete clinical examination, resting upper airway endoscopy was performed in all of the horses (*n* = 366). With this aim, the horses were contained in a stock; to prevent interferences with upper airway function, no other physical or pharmacological restraint techniques were applied. A flexible videoendoscope (EC-530WL-P, Fujifilm, Tokyo, Japan) was passed through the left nasal passage, and the upper tract of the respiratory system was visualized. A 0–4 score was assigned to pharyngeal lymphoid hyperplasia (PLH) [39] observed at the level of the nasopharyngeal mucosa and/or dorsal pharyngeal recess. The larynx was visualized, and its function was assessed during spontaneous breathing and after the stimulation of laryngeal movements by inducing swallowing, performing nasal occlusion maneuvers and during the “slap test” (thoraco-laryngeal reflex). Epiglottis conformation was evaluated, and its alterations were recorded, including the loss of rigidity and convexity (epiglottis flaccidity) or the presence of an entrapping mucosal fold. Whenever dorsal displacement of the soft palate was observed spontaneously or after the induction of swallowing, this finding was recorded. The observation of an ulceration on the free margin of the soft palate was registered. Based on arytenoids abductive function, a 1–4 score was assigned to recurrent laryngeal neuropathy (RLN) [40]. Whenever an alteration suggestive of a previous surgical treatment of the upper airway was observed (i.e., laryngoplasty, ventriculectomy, cordectomy, staphylectomy, etc.), the horse was excluded from the study (*n* = 6).

### 2.3. High-Speed Treadmill Endoscopy

Before performing high-speed treadmill endoscopy (HSTE), the horses were conditioned to the high-speed treadmill (Sato I, Uppsala, Sweden) by at least two training sessions. All the horses (*n* = 360) were tacked with the same equipment used for racing and wore a heart rate (HR) meter (Polar, Equine Inzone FT1, Steinhausen, Switzerland) during exercise. To perform HSTE, the horses were first warmed up by a 4-min walk (1.5 m/s) and a 5-min trot (4.5 m/s for Thoroughbreds, 6 m/s for Standardbreds), with a 6° slope for Thoroughbreds and 3° slope for Standardbreds. After warm-up, the treadmill was temporarily stopped, and a videoendoscope (ETM PVG-325, Storz, Tuttlingen, Germany) was passed into the nasopharynx of the horse and held in position with Velcro^®^ straps. Then, the treadmill was rapidly accelerated up to maximal speed (corresponding to HR ≥ 220 bpm) for a distance ranging from 1600 to 2100 m (based on the usual racing activity of each individual) or until the horse’s fatigue [41]. The endoscopic images were visualized in real-time on a monitor and recorded on analogic (from 2000 to 2004) or digital supports (from 2005 to 2021) to allow for slow-motion analysis and storage. All the registered videos were later evaluated by the same operator, and the absence or presence of single or multiple DUAOs (DDSP, NPC, MDAF, EE, ER, DLC) was recorded. Based on the entity of airway obstruction, the DUAOs were classified as severe (DDSP, NPC, DLC and ER) or mild (MDAF and EE) [7].

### 2.4. Radiographic Measurement of Epiglottis Length

**Figure 1:**
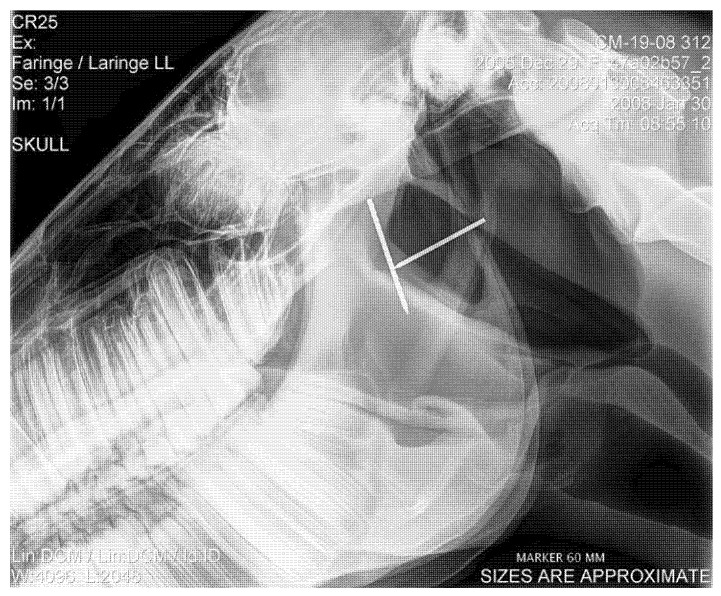
Example of how the radiographs were taken, with the markers of known length in place, to allow for epiglottis length measurements.

To measure the length of the epiglottis radiographically, a lateral view of the pharyngo-laryngeal region was taken on standing horses (*n* = 158), with the head placed in a normal resting position. Radiodense markers of a known length were fixed on both sides of the neck and superimposed to the nasopharynx or the guttural pouches (Figure 1). The lengths of the markers were measured on the obtained radiographs and were used to determine the grade of radiographical magnification. Thyroepiglottic length was then measured on the radiograph, and a correction factor for the previously assessed magnification was applied to obtain the actual epiglottis length [18]. The reference values for epiglottis length were considered to be 8.76 ± 0.44 cm for Thoroughbreds [18,42] and 8.74 ± 0.38 cm for Standardbreds [20,42,43].

### 2.5. Post-Exercise Tracheobronchoscopy

Thirty minutes after the end of the HSTE, a tracheobronchoscopy was performed to verify whether exercise-induced pulmonary hemorrhage (EIPH) had occurred. The horses (*n* = 339) were contained in a stock and restrained with a twitch; endoscopy was performed as described above, and the lower tract of the respiratory system was examined. A 0–4 score was assigned to the presence of blood in the trachea and the mainstem bronchi [44].

### 2.6. Tracheobronchoscopy at Rest and BALf Collection

At least 24 h after HSTE, an endoscopy of the lower airways was performed as described above. With this aim, the horses (*n* = 297) were sedated with detomidine hydrochloride (0.01 mg/kg IV) and restrained with a twitch. A 0–5 score was assigned to tracheal mucus accumulation (TM) [45]. The BALf was collected in 265 horses as follows: 60 mL of a 0.5% lidocaine hydrochloride solution was sprayed at the level of the carena to inhibit a coughing reflex, and the endoscope was passed into the bronchial tree until it was wedged firmly within a segmental bronchus; here, a 300 mL sterile saline 0.9% was instilled, and the fluid immediately aspirated [46].

### 2.7. BALf Cytological Examination

The collected BALf was stored in sterile EDTA tubes and processed within 90 min. A few drops of pooled BALf were cytocentrifugated (Rotofix 32, Hettich Cyto System, Tuttlingen, Germany) at 500 rpm for 5 min. The slides were air dried, stained with May–Grünwald Giemsa and Perl’s Prussian blue and observed under a light microscope at 400× and 1000× for 400-cell leukocyte differential count and the calculation of a simplified total hemosiderin score (THS) [47].

### 2.8. Statistical Analysis

The data were collected on an electronic sheet (Microsoft Excel, Redmond, WA, USA), analyzed with descriptive statistics and evaluated for normality by the Shapiro–Wilk test. Ages were compared between males and females and between Standardbreds and Thoroughbreds using the Mann–Whitney test. The associations between age and endoscopic scores (PLH, EIPH, TM), epiglottis length, BALf leukocyte populations and THS were evaluated by means of the Spearman correlation. The Mann–Whitney test was used to compare ages between horses with normal upper airways at rest and horses with any anatomical or functional alterations and between horses with and without DDSP at rest, epiglottis flaccidity and RLN. The same test was used to compare endoscopic scores, epiglottis lengths and BALf cytological findings between males and females and between Standardbreds and Thoroughbreds. The frequency of resting pharyngo-laryngeal alterations was compared between horses of different sexes and breeds using the Fisher’s exact test. The horses were divided into four groups on the basis of the HSTE findings: no-DUAO, mild-DUAO, severe-DUAO or multiple-DUAOs. Ages was compared between the groups using the Kruskal–Wallis test and Dunn’s multiple comparisons test; the distribution of sex and breed was compared between the groups by the Chi-square test. The Kruskal–Wallis test and the Dunn’s multiple comparisons test were used to compare the endoscopic scores, epiglottis lengths, and BALf cytological results between the groups. The PLH score was also compared between the no-DUAO horses and the horses with dynamic DDSP, the horses with NPC and the horses with MDAF by the Mann–Whitney test; the same test was used to compare epiglottis lengths between the no-DUAO horses and the horses with dynamic DDSP. The frequency of pharyngo-laryngeal alterations at rest was compared between the groups by means of the Chi-square test and between the horses without DUAOs and the horses with dynamic DDSP, the horses with NPC and the horses with MDAF using the Fisher’s exact test. The resting RLN grade was compared between the horses with and without DLC by means of the Mann–Whitney test. Moreover, the PLH score was compared between the horses with normal upper airway at rest and the horses with resting DDSP and between the horses with normal resting endoscopy and the horses with epiglottis flaccidity using the Mann–Whitney test. Finally, the frequency of DDSP at rest was compared between the horses with normal epiglottis and the horses with epiglottis flaccidity by means of the Fisher’s exact test. The data are presented as the mean ± standard deviation (SD) if normally distributed and as the median and interquartile ranges (IQRs) if not normally distributed. The statistical significance was set at *p* < 0.05. The data were analyzed using a commercially available statistical software package (GraphPad Prism 9.3.1 for MacOS; GraphPad Software, San Diego, CA, USA).

## 3. Results

### 3.1. Horses

A total of 360 horses (312 Standardbreds and 48 Thoroughbreds) met the inclusion criteria. The population consisted of 233 males (203 stallions, 30 geldings) and 127 females, aged from 2 to 8 years (median 3 years, IQR 3–4 years). Among the Standardbred population, 200 horses were males (64.1%) and 112 were females (35.9%), while, among the Thoroughbred population, 33 subjects were males (68.75%) and 15 were females (31.25%). The median age was 3 years in both the Standardbred and Thoroughbred populations, with different IQRs (Standardbreds: IQR 3–4 years; Thoroughbreds: IQR 3–3 years). The horses were divided into the no-DUAO group (169 horses), mild-DUAO group (23 horses), severe-DUAO group (111 horses) and multiple-DUAOs group (57 horses).

### 3.2. Upper Airway Endoscopy at Rest

The frequency and grade distribution of PLH among the study population are shown in Table 1; the median PLH score was 2 (IQR 1–2). The normal appearance and function of the pharyngo-laryngeal region were observed in 160 horses (44.44%), while alterations were detected in 200 horses (55.56%); the distribution of upper airway alterations detected at resting endoscopy is displayed in Table 2.

### 3.3. High-Speed Treadmill Endoscopy

During HSTE, no DUAO occurred in 169 horses (46.95%), one single DUAO was observed in 134 horses (37.22%) and multiple concomitant DUAOs were detected in 57 horses (15.83%). In order of frequency, the observed DUAOs included DDSP (107 horses, 29.72%), MDAF (63 horses, 17.5%), NPC (59 horses, 16.39%), DLC (19 horses, 5.28%), EE (7 horses, 1.94%) and ER (2 horses, 0.56%). The frequency of single or concomitant DUAOs is displayed in Table 3.

### 3.4. Epiglottis Length

The epiglottis length was radiographically measured in 145 Standardbreds and 13 Thoroughbreds. The median epiglottis length in the investigated population was 8.2 cm (Standardbreds: IQR 7.88–8.7 cm; Thoroughbreds: IQR 7.5–8.8 cm). In the Standardbred population, the epiglottis length was within the reference ranges in 60 horses (41.67%), lower in 73 horses (50.69%) and higher in 11 horses (7.64%); in the Thoroughbreds population, the epiglottis length was within the normal limits in 2 horses (15.38%), lower in 8 horses (61.54%) and higher in 3 horses (23.08%).

### 3.5. Lower Airway Endoscopy and BALf Cytology

The tracheobronchoscopy performed 30 min after maximal exercise was negative for EIPH in 145 horses (42.77%) and positive in 194 horses (57.23%); the frequency and grade distributions of EIPH among the study population are shown in Table 4. The median EIPH grade was 1 (IQR 0–2). On the lower airway endoscopy performed at least after 24 h, tracheal mucus accumulation was observed in 296 horses (86.6%); the grade distribution is displayed in Table 5. The median TM score was 1 (IQR 1–2). The cytological examination of BALf showed a median of 46% (40–50.5%) macrophages, a mean of 35.72% ± 11.85% lymphocytes, a median of 10% (5–18%) neutrophils, a median of 1% (0–3%) eosinophils and a median of 4% (3–6%) mast cells. The median THS was 36 (12–66).

### 3.6. Influence of Age, Breed and Sex

Among the study population, the Thoroughbreds were younger than the Standardbreds (*p* = 0.0054), and the females were younger than the males (*p* < 0.0001). Sex was equally distributed among the different breeds. Age was inversely correlated with PLH grade (*p* < 0.0001, r = −0.45) and TM score (*p* = 0.0083, r = −0.14) and was positively correlated with EIPH score (*p* = 0.0053, r = 0.15) and THS (*p* = 0.0165, r = 0.15); PLH score was significantly higher in females (median 2, IQR 2–3) than in males (median 2, IQR 1–2) (*p* < 0.0001). The horses showing pharyngo-laryngeal alterations at resting endoscopy were younger than the horses with normal appearance and function (*p* = 0.0213); moreover, pharyngo-laryngeal alterations were significantly more frequent in Thoroughbreds (83.33%) than in Standardbreds (51.28%) (*p* < 0.0001). In particular, DDSP at rest and grade > 1 RLN was more frequently observed in Thoroughbreds (DDSP 47.92%, RLN 63.83%) than in Standardbreds (DDSP 24.04%, RLN 25.32%) (DDSP *p* = 0.0014; RLN *p* < 0.0001). At BALf cytology, the neutrophils counts were higher in Thoroughbreds (median 17%, IQR 11–22.5%) than in Standardbreds (median 9%, IQR 4–17%) (*p* = 0.0016), while the lymphocyte counts were higher in Standardbreds (36.25% ± 11.78%) than in Thoroughbreds (30.92% ± 11.63%) (*p* = 0.0293). Among the different groups (no-DUAO, mild-DUAO, severe-DUAO and multiple-DUAOs), no differences were observed in age and sex distribution, while a significant difference in breed distribution was detected (*p* = 0.0064); in particular, in Thoroughbreds, DUAOs were significantly more frequent than they were in Standardbreds (Thoroughbreds 75%, Standardbreds 50.32%; *p* = 0.001).

### 3.7. Upper Airway Endoscopy and Epiglottis Length vs. DUAOs

**Figure 2:**
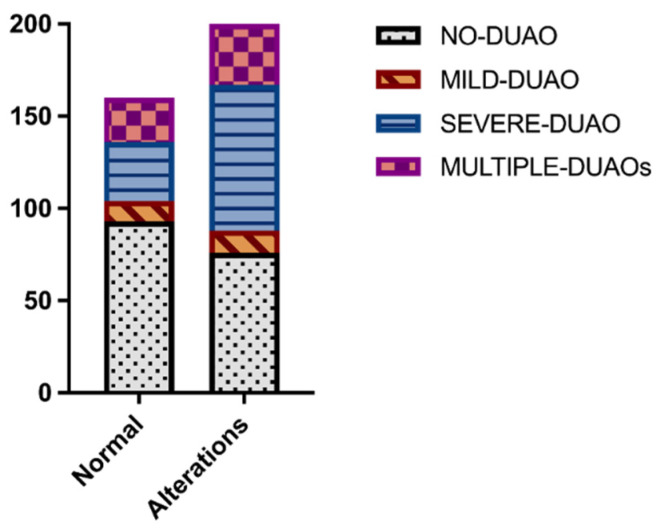
Stacked bars showing the frequency of alterations at upper airway resting endoscopy in the different groups (no-DUAO, mild-DUAO, severe-DUAO and multiple-DUAOs).

**Figure 3:**
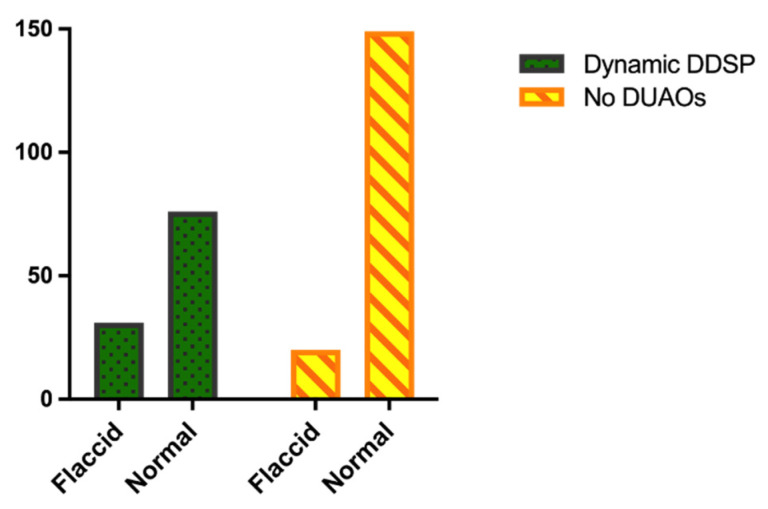
Bar graph showing the frequency of normal or flaccid appearance of epiglottis at upper airway resting endoscopy in the horses with no DUAOs and with DDSP diagnosed at HSTE.

The PLH grade was significantly lower in the multiple-DUAOs group (median 1, IQR 0–2) compared to the no-DUAO group (median 2, IQR 1–3) (*p* = 0.0066) and the severe-DUAO group (median 2, IQR 1–2). No differences were observed in PLH between the horses with and without DDSP, MDAF and NPC. The epiglottis length did not differ between the groups, nor did it differ between the horses with and without DDSP. The frequency of abnormalities of pharyngo-laryngeal appearance and function detected at resting endoscopy differed between the groups (*p* = 0.0003) (Figure 2); in particular, alterations were more frequently observed in the severe-DUAO group (71.17%) compared to the no-DUAO group (44.97%) (*p* < 0.0001). While no differences were observed between the groups regarding the frequency of DDSP episodes observed during resting endoscopy, a trend was detected for different frequencies of epiglottis flaccidity among the different groups (*p* = 0.0534). In particular, a flaccid appearance of epiglottis was more frequently observed in the horses with DDSP diagnosed at HSTE (60.78%) compared to the horses without DDSP (39.22%) (*p* = 0.0007) (Figure 3). No differences were observed in the frequency of DDSP at rest between the horses with and without DDSP during HSTE, nor in the frequency of resting DDSP and epiglottis flaccidity between the horses with and without NPC and MDAF. The horses showing DDSP episodes at rest had a higher PLH grade (median 2, IQR 1–3) compared to the horses without resting DDSP (median 2, IQR 1–2) (*p* = 0.0052) and more frequently showed a flaccid appearance of the epiglottis (resting DDSP 31.63%, no resting DDSP 12.21%, *p* < 0.0001). Among the seven horses showing EE during exercise, three also presented persistent EE visible at resting endoscopy, two had a normal epiglottis and two had a flaccid appearance of the epiglottis; in four cases, an ulceration of the free margin of the soft palate was observed. Ulcerations of the margin of the soft palate were also observed in the four horses without DUAO, the two horses with MDAF + NPC, the two horses with DDSP and the two horses with NPC + DDSP. In the horses with DLC, the RLN grade was significantly higher (median 3, IQR 3–3) compared to the horses with normal dynamic arytenoids abduction (median 1, IQR 1–2) (*p* < 0.0001). In particular, all the horses with grade 1 (normal) RLN showed a normal laryngeal abduction during HSTE, and among the horses with grade 2 RLN, only two horses showed DLC (2.15%); in contrast, all the horses with grade 3 and grade 4 RLN showed DLC on HSTE.

### 3.8. Lower Airway Endoscopy and BALf Cytology vs. DUAOs

No differences between the groups were observed regarding the TM score, EIPH score, leukocyte differential count of BALf or THS.

## 4. Discussion

Although DUAOs are disorders that have long been known to cause poor performance in racehorses, their precise pathogenetic mechanisms have not been fully understood yet, and the possible role of structural or functional abnormalities of the nasopharynx has not been univocally clarified [8,9,10,11]. Moreover, some authors suggest that inflammation of the upper and lower respiratory tract may contribute to the development of DUAO, but contrasting results have been reported [26,27,28,29,30,31,32,33]. Therefore, the present study aimed to investigate the diagnostic role of upper and lower airways resting evaluation for a better understanding of DUAO development.

The value of this study relies on a very large and homogeneous population of racehorses, including a total of 360 patients, and on a highly standardized protocol for airways evaluation in both resting and dynamic conditions. Among the study population, 53.05% of the horses showed DUAO on HSTE; the prevalence of DUAO reported in the literature ranges from 22.6% to 80.1% [48,49], based on the kind of population (i.e., racehorses or other performance horses), inclusion criteria (randomized or horses with a history of poor performance and respiratory noises) and diagnostic technique (HSTE or overground endoscopy). Among reports including both Standardbreds and Thoroughbreds diagnosed by HSTE, DUAO prevalence varies from 22.6% [48] to 65% [22]. Interestingly, the highest frequencies have been reported by studies only including Thoroughbred racehorses, with percentages ranging from 72.6% to 80.1% [5,49,50,51]; similarly, in our study 75% of the Thoroughbred patients showed DUAO during HSTE, which was significantly more frequent than in Standardbreds (50.32%), suggesting a breed predisposition. However, in our population, most Standardbreds were referred for poor performance, while Thoroughbreds were more often referred for abnormal respiratory noises during exercise, with a consequent higher probability of being diagnosed with DUAO; this could have biased the results of our study. An apparent predisposition of Thoroughbreds to develop DUAO, and especially DDSP, in comparison with Standardbreds was also observed by previous researchers, but the reason for this breed discrepancy is unclear [11,52,53]. In our study, the Thoroughbreds more commonly showed upper respiratory tract alterations at rest; it must be noticed that the Thoroughbreds were younger than the Standardbreds, probably due to the shorter racing career, and that alterations at rest were more frequently observed in younger horses. Some authors suggest that young age may predispose horses to the development of DUAO [5,9] and that affected horses could spontaneously resolve this condition over time [10]; in fact, it has been hypothesized that increased palatal musculature in older horses may improve muscular activity and provide a better resistance against collapsing forces [5]. In contrast, other reports found no association between age and DUAO [11] or even a higher susceptibility in older horses [54].

In the present study, the most frequently observed forms of DUAO were DDSP (29.72%), MDAF (17.5%) and NPC (16.39%); likewise, most studies about racehorses report DDSP as the most common DUAO, with the prevalence ranging from 14% to 45.2% [5,14,22,48,49,50,51,55]. MDAF was the most common DUAO in a previous study [11], but its prevalence in the literature is highly variable, ranging from 4% to 40.4% [5,11,22,49,50,51]. Both in the present study and in the previous reports, MDAF is rarely diagnosed alone and is associated, in most cases, with other types of DUAO, especially NPC and DDSP; therefore, various authors have suggested that MDAF may either contribute or follow the development of associated DUAOs [5,8,11,12,38]. More generally, multiple concomitant DUAOs were detected relatively frequently in our population, with a prevalence of 15.83% of the total horses and of 29.84% of the horses with DUAO. Multiple DUAOs have also been commonly reported by previous studies, with a wide range of prevalence varying from 7% to 56.9% [4,5,11,15,22,49,50]. In the present report, NPC showed a prevalence of 16.39%, which was similar to those reported by some authors [4,14,50,51] but quite higher when compared to other studies [5,11,22,23,49]. The great variability of the prevalence of DUAOs, as reported by different studies, may be attributable to heterogeneous enrolled populations, diagnostic techniques and the lack of a consensus statement which could provide universal guidelines for the classification of different DUAOs [56]. The only upper respiratory tract for which a consensus statement exists is RLN detectable at rest [40]; in the present study, all of the horses with grade 1 RLN and 97.85% of the horses with grade 2 RLN showed a normal laryngeal function during exercise. In contrast, some authors have reported that low percentages (from 0.34% to 3.5%) of horses with grade 1 RLN and up to 11.9% of horses with grade 2 RLN may show DLC on HSTE [2,23,57]. Moreover, in our study, all horses with grade 3 and grade 4 RLN developed DLC on HSTE; conversely, previous studies reported a normal laryngeal function during exercise in 16% to 33% of the horses with grade 3 RLN [23,57], and it has been reported that 2% of horses with grade 4 RLN may still show a residual laryngeal activity during exercise without experiencing DLC [57]. Therefore, independently from the RLN grade at rest, HSTE should be always encouraged to evaluate dynamic laryngeal function.

It has been hypothesized that epiglottic hypoplasia may predispose horses to DUAOs, such as DDSP, MDAF and EE [12,13,14,15,18]; in the present study, epiglottis length was assessed radiographically, and alterations of its conformation visible at endoscopic examination were recorded. Interestingly, only 39.49% of our patients had a normal epiglottis length falling within the reference ranges [18,20,42,43]; a shorter epiglottis was observed in 51.59%, while the remaining 8.92% had a longer epiglottis. As the technique used for radiographic measurement was the same as that described by Linford (1983) [18], and our population of Standardbreds is by far the widest that underwent radiological assessment of epiglottis length, it is possible that the reference ranges may be reviewed, including more numerous populations of Standardbreds and Thoroughbreds. Unfortunately, only 158 horses in the present study underwent epiglottis length measurement, of which 145 were Standardbreds; the low number of Thoroughbreds subjected to this measurement represents a main limitation for the evaluation of the possible relationship between epiglottis length and the development of DUAOs. In our study epiglottis length did not influence the development of DUAO or the development of any resting alterations; this finding is in contrast with a previous one reporting a shorter epiglottis as a predisposing factor to DDSP [18]. However, similarly to our findings, other studies failed to detect any association between epiglottis length and DUAO [9,19]. Therefore, some authors suggested that the epiglottis may play a role in DUAO development—not for its length but for its conformation. In particular, the loss of rigidity and of the normal convex appearance has been associated with DDSP and MDAF [12,58]. Our findings partially confirm this hypothesis: among our population, the horses showing a flaccid appearance of epiglottis at endoscopic examination were more prone to develop DDSP both at rest and during exercise, while no relationship was observed with MDAF. As alterations at resting endoscopy were more frequent in the severe-DUAO group, we also evaluated whether DDSP detected at rest could be a good predictor of DDSP on HSTE; however, no association between DDSP at rest and dynamic DDSP was observed. Conversely, previous studies reported a low sensitivity but a high specificity of DDSP detected at rest [2,22,27]. In any case, upper respiratory tract endoscopy at rest is advised in cases of suspected DUAO, but dynamic endoscopy is confirmed to always be essential for a certain diagnosis.

It has been hypothesized that inflammation of the upper airways may predispose horses to DUAO [8,9,25,26,27,28,29,30]. In particular, PLH has been associated with DDSP [9,27,28], NPC [29,30] and ER [8]. Surprisingly, in our study, PLH was lower in horses belonging to the multiple-DUAOs group; a possible explanation for this may be that the horses in the multiple-DUAOs group were older than those in the other groups, even if a statistical significance was not detected. As the PLH grade was lower in older horses, a lower PLH grade in the multiple-DUAOs group may ensue, without implying any causative relation between a low PLH grade and the concomitant occurrence of multiple DUAOs. When individually evaluating different DUAOs, no association was detected between PLH and DDSP, MDAF or NPC. Unfortunately, in our study, it was not possible to individually evaluate the other types of DUAOs due to the low number of affected cases. Interestingly, the PLH grade was higher in horses experiencing DDSP at rest but not during exercise. It could be hypothesized that upper airway inflammation may have an influence on pharyngo-laryngeal movements at rest; however, during exercise, more forces come into play, and the contribution of pharyngeal inflammation may be too low to have an impact on pharyngeal stability.

Some authors have also reported an association between DUAO and LAI [9,28,30,31,32,33]; in particular, a relation between BALf neutrophilia and DDSP was reported in two studies from the same research group [9,28], while other studies detected an association between mild-moderate equine asthma and NPC [30,33]. However, multiple studies reported no association between upper and lower airways inflammation based on endoscopic and cytological findings [46,59,60,61], and other authors found no relationship between LAI and DUAO [38]. Similarly, in the present study, no differences between the groups were observed concerning tracheal mucus accumulation or the BALf cytological profile, suggesting that LAI is not associated with the onset of DUAO. Another lower airway disorder which has been associated with DUAO is EIPH [1,31,36,37]; however, analogously to a previous study [38], we found no relationship between DUAO and EIPH based on both post-exercise endoscopy and total hemosiderin score. Therefore, the findings of the present study do not support the theory of a possible contribution of upper and lower airways inflammation to the onset of DUAO, nor the cause–effect relationship between DUAO and EIPH; however, given the contrasting results reported by different studies, these hypotheses cannot be ruled out.

## 5. Conclusions

In conclusion, DUAOs were more commonly observed in Thoroughbred racehorses than in Standardbred racehorses, confirming a possible breed predisposition; conversely, age did not seem to influence the onset of DUAO on HSTE, as pharyngo-laryngeal alterations were detected more frequently in younger horses only at resting upper airway endoscopy. The detection, at resting endoscopy, of the abnormal appearance and function of the pharyngo-laryngeal region was associated with the development of DUAO: in particular, a flaccid appearance of the epiglottis, with a loss of convexity and rigidity, was associated with the occurrence of DDSP both at rest and on HSTE. In contrast, the epiglottis length measured radiographically was not associated with DUAO, suggesting that the epiglottis may be involved in maintaining the stability of the upper respiratory tract, not based on its dimensions but on its conformation. Finally, in the present study, no association was observed between DUAO and the inflammation of the upper and lower airways, nor between DUAO and EIPH.

## Figures and Tables

**Table 1:** Frequency and distribution of pharyngeal lymphoid hyperplasia (PLH) grades among the study population.

PLH Grade	% of the Study Population
0	16.67%
1	17.22%
2	43.89%
3	20.83%
4	1.39%

**Table 2:** Distribution of upper airways endoscopic findings at rest.

Endoscopic Finding	% of the Study Population
Normal appearance and function	44.44%
Recurrent Laryngeal Neuropathy	30.27% 25.83% Grade II, 3.33% Grade III (0.28% III.a, 1.38% III.b, 1.67% III.c), 1.11% Grade IV
One or more episodes of dorsal displacement of the soft palate	27.22%
Epiglottis flaccidity	17.5%
Ulceration of the free margin of the soft palate	3.89%
Persistent epiglottis entrapment	1.11%

**Table 3:** Frequency of single or multiple dynamic upper airway obstructions (DUAOs) detected during high-speed treadmill endoscopy. DDSP = dorsal displacement of the soft palate; NPC = nasopharyngeal collapse; MDAF = medial deviation of aryepiglottic folds; DLC = dynamic laryngeal collapse; EE = epiglottis entrapment; ER = epiglottis retroversion.

DUAOs	% of the Study Population
DDSP	20.83%
NPC	7.22%
MDAF	6.11%
DLC	2.5%
EE	0.28%
ER	0.28%
MDAF + NPC	5.28%
MDAF + DDSP	2.22%
NPC + DDSP	1.94%
DDSP + EE	1.67%
MDAF + DLC	1.39%
DDSP + DLC	0.83%
MDAF + ER	0.28%
MDAF + NPC + DDSP	1.67%
MDAF + DDSP + DLC	0.28%
MDAF + NPC + DDSP + DLC	0.28%

**Table 4:** Frequency and grade distribution of exercise-induced pulmonary hemorrhage (EIPH) among the study population.

EIPH Grade	Number of Horses	% of the Study Population
0	145	42.77%
1	75	22.12%
2	74	21.83%
3	34	10.03%
4	11	3.25%

**Table 5:** Frequency and grade distribution of tracheal mucus accumulation (TM) among the study population.

TM Grade	Number of Horses	% of the Study Population
0	45	13.2%
1	142	41.64%
2	117	34.31%
3	33	9.68%
4	4	1.17%

## Data Availability

The data presented in this study are available on request from the corresponding author.
